# Current Status of CT Imaging Before Common Transcatheter Interventions for Structural Heart Disease

**DOI:** 10.3390/diagnostics15010097

**Published:** 2025-01-03

**Authors:** Rodrigo Salgado, Farah Cadour, Riccardo Cau, Luca Saba

**Affiliations:** 1 Department of Radiology, Antwerp University Hospital, Drie Eikenstraat 655, 2650 Edegem, Belgium; 2 Faculty of Medicine & Health Sciences, University of Antwerp, Universiteitsplein 10, 2610 Wilrijk, Belgium; 3 Department of Radiology, Heilig Hart Ziekenhuis Lier, Mechelsestraat 24, 2500 Lier, Belgium; 4 Department of Medical Imaging, University of Toronto-University Medical Imaging Toronto, UHN, 585 University Ave, Toronto, ON M5G 2N2, Canada; farah.cadour@hotmail.fr; 5 Department of Radiology, Azienda Ospedaliero Universitaria, University of Cagliari, 09124 Cagliari, Italy; riccardocau00@gmail.com (R.C.); lucasabamd@gmail.com (L.S.)

**Keywords:** TAVR, TAVI, pulmonary vein ablation, CT, transcatheter, atrial septal defect, left atrial appendage closure

## Abstract

**Background:** Over the past decade, several trials and observational studies have validated the use of minimally invasive cardiac interventions as viable treatment options for various cardiac diseases. Transcatheter techniques for severe aortic valve stenosis have rapidly emerged as alternatives to surgical aortic valve replacement in certain patient populations. Additionally, non-surgical treatment options have expanded for conditions affecting other cardiac valves, such as the mitral valve. These emerging minimally invasive interventions complement already well-established endovascular techniques for, among others, atrial septal defect closure, left atrial appendage occlusion and pulmonary vein isolation in patients with atrial fibrillation. Given their non-surgical nature and lack of direct visualisation of the targeted anatomy, these procedures heavily rely on precise pre-procedural radiological imaging for optimal patient selection and procedural success. **Method:** This paper is based on the expert opinion of the authors and an exhaustive literature research. **Results:** This manuscript reviews the most commonly employed minimally invasive cardiac interventions, highlighting the essential pre-procedural imaging information and key aspects that must be included in radiological reports to mitigate potential complications. **Conclusion:** Accurate pre-procedural imaging is crucial for ensuring safe and effective minimally invasive cardiac interventions, underscoring the importance of the radiologist in the pre-procedural work-up of these patients.

## 1. Introduction

For decades, surgery has been the method of choice for the treatment of a variety of structural heart diseases once optimal medical therapy and other non-surgical interventions have failed to provide a definite solution. However, while cardiac surgery remains in many instances the treatment of choice, in the last decade, we have seen an increase in the use of transcatheter techniques to treat a variety of cardiac conditions. These techniques have been developed either as additional therapeutical options for specific patient populations which are ineligible for cardiac surgery (e.g., severe aortic stenosis in elderly patients) or as further non-surgical alternatives to already established procedures applicable to all patients (e.g., left atrial appendage closure).

These cardiac interventions increasingly rely on accurate pre-procedural measurements to obtain detailed knowledge of the patient’s anatomy, the dimensions of the target structure and any other potential obstructions along the delivery route. These are key points to ensure a successful procedural outcome.

Therefore, computed tomography (CT) has become a cornerstone of the pre-procedural work-up in many of these techniques. This paper explores the advancements in pre-interventional measurement techniques using CT, highlighting its impact on these procedures.

## 2. Atrial Septal Defects

The embryology of atrial septal defects (ASDs) has been extensively described elsewhere [1]. In summary, during foetal development, the atrial septum starts as a single layer of tissue known as the atrial septum primum. Afterwards, a small septal opening called the ostium primum forms in the atrial septum primum, allowing for interatrial blood flow. Subsequently, a second layer of tissue, the atrial septum secundum, develops alongside the atrial septum primum, partially covering the ostium primum. The ostium secundum emerges as a second opening in the atrial septum secundum. Typically, the ostium secundum and ostium primum eventually merge, closing the gap between the atria. However, fusion failure will lead to the development of atrial septal defects. The specific location and extent of fusion contribute to the variability in the size of these defects and the differences observed within the same classification of defects.

**Figure 1:**
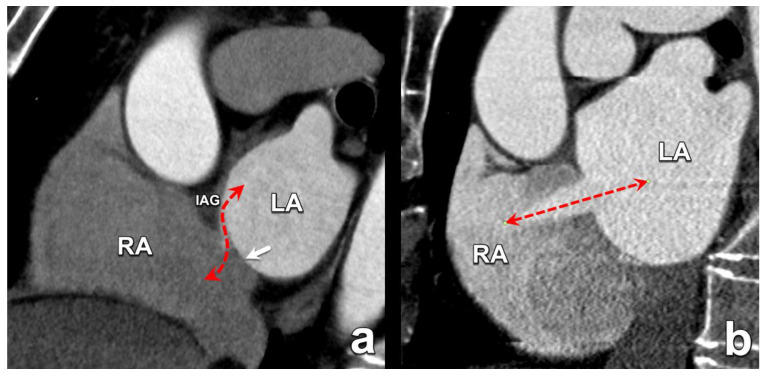
The different embryological origins of PFO and ASD are reflected in their morphological appearance. A PFO is the result of failed fusion between the interatrial groove (IAG) and the septum primum (arrow), resulting in an intermittent interatrial communication with an oblique trajectory between the atrial septal rims (dashed line in (**a**)). Conversely, an ASD is a permanent connection between the left and right atrium with a trajectory perpendicular to the axis of the interatrial septum and without overlapping septal rims. (dashed line in (**b**)). LA: left atrium; RA: right atrium; ASD: atrial septal defect; PFO: patent foramen ovale.

An ASD must be differentiated from a patent foramen ovale, which is more prevalent in the general population and seldom poses a clinical problem (Figure 1). While many patients with an ASD can remain asymptomatic for prolonged periods of time, often not being diagnosed during childhood, many will become symptomatic starting in the fifth decade of life. As the interatrial shunt persists with chronic right heart volume overload, advanced age will increasingly lead to exercise intolerance, atrial fibrillation, decompensated right heart failure and pulmonary hypertension [2].

Not every interatrial communication is categorised as an ASD. Table 1 provides an overview of possible interatrial communications with their clinical consequences and preferred treatments.

Surgical closure remains the approach of choice for sinus venosus defects, ostium primum ASDs and coronary sinus defects [3]. Nevertheless, advances in transcatheter techniques have largely replaced previous surgical approaches to repair ostium secundum ASDs, pending suitable anatomy. A transcatheter approach, in general, allows for a shorter hospital stay and lower procedure-related morbidity and mortality [4]. However, despite the development of many septal closure devices, surgery still remains in some cases the only feasible option for certain conditions: a large secundum ASD > 36 mm; inadequate atrial septal rims (defined as the tissue between the defect and adjacent structures) which do not allow for stable device deployment; or proximity of the defect to the atrioventricular valves, the coronary sinus or the venae cavae [5].

The indication for intervention remains the same for either approach, mostly based on signs of right atrial and right ventricular dilation secondary to volume overload.

In the absence of an intracardiac shunt, the effective pulmonary blood flow (Qp) is equivalent to the effective systemic blood flow (Qs), maintaining a Qp/Qs ratio of 1:1. However, a hemodynamically significant ASD induces an increase in pulmonary blood flow, resulting in an elevated Qp/Qs ratio. The magnitude of this left-to-right shunt, quantified by the Qp/Qs ratio, serves as a critical parameter in determining the need for intervention.

Current clinical guidelines stipulate that a Qp/Qs ratio exceeding 1.5:1 is an important criterion for recommending either surgical or transcatheter ASD closure. This threshold is based on hemodynamic principles and clinical outcome data, which indicate that patients with a Qp/Qs ratio below 1.5:1 are less likely to derive a significant benefit from defect closure, either in terms of symptom improvement or long-term cardiovascular outcomes [6].

In practice, the determination of the Qp/Qs ratio typically involves CMR, allowing for the precise quantification of blood flow dynamics and shunt magnitude and enabling evidence-based clinical decision-making regarding ASD management.

The two main components that must be evaluated in a candidate for transcatheter septal closure are the size of the ASD and the size of the adjacent septal rims. Table 2 provides an overview of several available closure devices and their requirements for the targeted septal defect. Measurements of the ASD should be performed in multiple planes. Mostly, an ASD will not be circular but have a more oval morphology, with short and long axes. Given the excellent volumetric dataset of CT, the area of the defect can be easily measured. However, as manufacturers’ guidelines are often based on echocardiography measurements, only one dimension is given for further reference. Following this guideline, most devices can close defects ranging from about 5 mm to 40 mm in diameter. The Amplatzer Septal Occluder, for example, is available in sizes from 4 mm to 38 mm. For smaller defects under 5 mm, the benefits of closure may not outweigh the risks of the procedure, so these are often monitored rather than closed, unless the patient is significantly symptomatic. Defects over 40 mm in diameter are more challenging, as larger devices required carry higher risks [5]. As stated before, for very large defects, surgical closure is often preferred over septal occluder devices.

**Figure 2:**
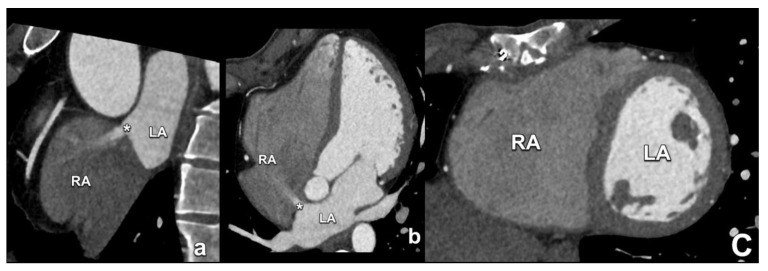
Ostium secundum ASD (asterisk in (**a**,**b**)) with inadequate septal rim, making this patient ineligible for transcatheter closure. Note the dilatation of the right ventricle due to chronic volume overload (**c**). RA; right atrium; LA: left atrium.

Regarding the size of the septal rim, in general, at least 5 mm of septal tissue is required to ensure stable device deployment and avoid embolisation [5,7,8] (Figure 2). In some cases, a deficient retroaortic rim may still allow for transcatheter closure, but this is evaluated on a case-by-case basis.

## 3. Transcatheter Aortic Valve Replacement

Calcific aortic stenosis (AS) represents the predominant aetiology of aortic stenosis globally and ranks as the third most prevalent cardiovascular pathology in developed nations, following coronary artery disease and hypertension [9]. The epidemiology of CAS is characterised by a sharp age-dependent increase in prevalence, affecting approximately 0.4% of the general population, with a significant rise to 1.7% in individuals over 65 years of age and a further escalation to 4.6% in those exceeding 75 years [10]. Demographic projections and the current lack of effective preventive strategies suggest that the burden of CAS is expected to increase by a factor of 2–3 over the next five decades in developed countries, primarily due to population ageing [9].

**Figure 3:**
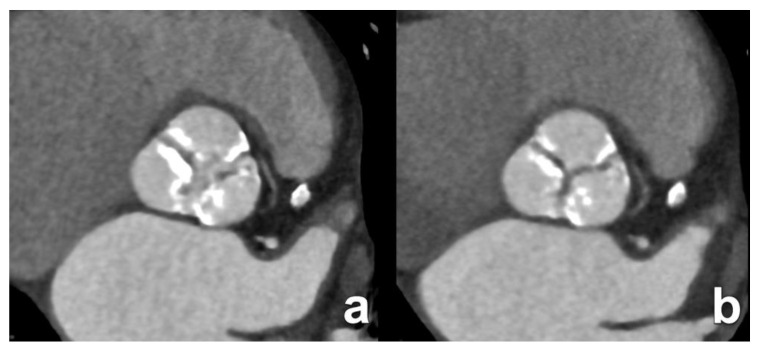
An 82-year-old man with severe symptomatic aortic stenosis. Double-oblique cross-sectional images through the aortic sinus in the systolic (**a**) and diastolic (**b**) phase. Note the thickened and heavily calcified leaflets in this tricuspid valve and the stenotic luminal area in systole (**a**).

In the geriatric population, AS typically manifests as a degenerative process characterised by valve leaflet thickening, fibrosis and extensive calcification (Figure 3). Conversely, in younger patient populations, the aetiology of AS often involves congenital malformations, with a bicuspid aortic valve being a significant predisposing factor that warrants consideration [11].

The pathophysiology of AS is marked by its progressive nature, evolving through distinct stages of severity. Clinical data suggest that approximately 10–15% of patients with moderate AS progress to severe AS annually [6]. The natural history of critical, symptomatic AS is associated with a poor prognosis, characterised by a median survival of approximately two years in the absence of intervention, translating to a 50% two-year mortality rate [12].

Surgical aortic valve replacement remains the gold standard of treatment for severe symptomatic AS [6]. However, the demographic profile of some patients with AS is characterised by a (very) advanced age and a concomitant high prevalence of comorbidities, which can significantly elevate perioperative risk and render some patients ineligible for traditional open-heart surgery.

In response to this challenge, the past decade has witnessed the emergence of transcatheter aortic valve replacement (TAVR) as an alternative therapeutic modality. TAVR offers a less invasive approach for high-risk and inoperable patients with severe AS, providing a new therapeutical option.

The fundamental principle of TAVR is as follows. The procedure primarily utilises the femoral artery as the preferred access route for device delivery, although other access routes exist to maximise patient eligibility. A transcatheter heart valve (THV) is advanced to the aortic sinus and deployed using either a balloon-expandable or self-expanding system, contingent upon the specific device manufacturer. The deployment process results in the compression of the native valve leaflets between the expanding THV and the aortic sinus wall. Upon full deployment, the THV assumes the functional role of the native valve, effectively restoring valvular competence.

Both balloon-expandable and self-expandable valves are available, with the Sapien series from Edward Lifesciences and the Evolut range from Medtronic being prime examples of each. Due to their deployment technique, self-expanding valves will mostly assume an oval cross-sectional shape after deployment, as they adhere to the native anatomy at the annual level [13]. Conversely, the radial force exerted by the balloon will force the circular deployment of balloon-expandable valves. This difference in deployment and final morphology is one reason that sizing algorithms for one valve cannot be used on a different valve. All valves are designed to exhibit some degree of oversizing to promote stability.

In contrast to conventional surgical approaches, TAVR evidently precludes a direct visual assessment of the aortic root anatomy. Consequently, intraoperative sizing and the selection of optimal THV dimensions are not feasible. This limitation necessitates the preoperative determination of THV size through detailed planimetric analysis of the aortic root, encompassing multiple levels from the sinotubular junction to the aortic annulus.

While echocardiography was initially employed in the seminal trials that validated TAVR for clinical use in non-operable and high-surgical-risk cohorts, computed tomography (CT) has emerged as the gold-standard imaging modality for preoperative planning [14,15]. CT imaging not only facilitates the precise visualisation and quantification of aortic root dimensions but also allows for a comprehensive evaluation of potential vascular access routes to identify any anatomical barriers that might impede THV delivery.

This imaging-guided approach to TAVR underscores the critical importance of advanced radiological techniques in optimising procedural outcomes and mitigating potential complications associated with device sizing or vascular access challenges.

**Figure 4:**
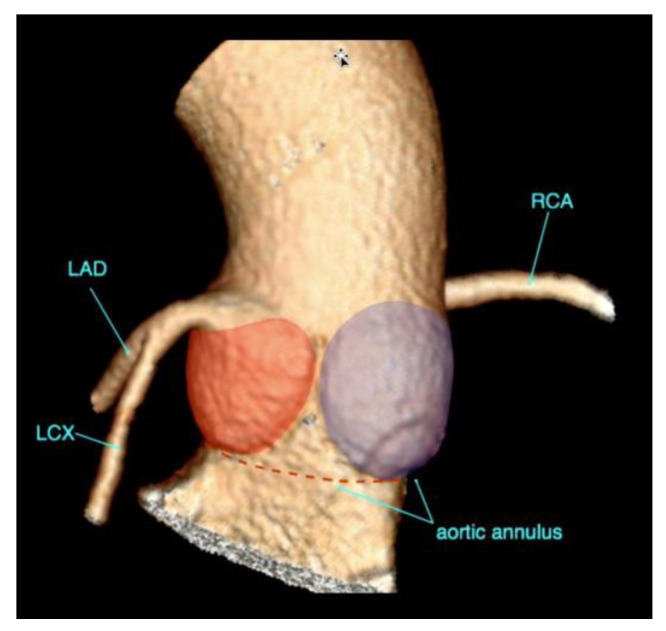
Volume-rendered image of the aortic sinus illustrating the aortic annulus. The annulus is a virtual structure, formed by connecting the nadirs of the insertion points of the aortic leaflets (dotted line). The left and non-coronary sinus of Valsalva are indicated in red and purple.LAD: left anterior descendens artery; RCA: right coronary artery; LCX Left circumflex artery.

One of the most important anatomical landmarks to scrutinise in a TAVR candidate is the aortic annulus. The aortic annulus is not a real anatomical structure but a virtual ring formed by connecting the nadirs of the most basal insertion points of the aortic valve leaflets (Figure 4). At this level, the annulus appears on cross-sectional imaging in many instances as an oval-shaped structure with short and long axes.

This is an important fact to consider when comparing imaging modalities, as echocardiography-derived measurements will usually return only one dimension (in practice, the largest diameter that can be obtained at the level of the aortic annulus), while CT will report two dimensions (long and short axes). This makes the direct comparison of echocardiography- and CT-derived measurements complicated, and it must be approached with caution.

**Figure 5:**
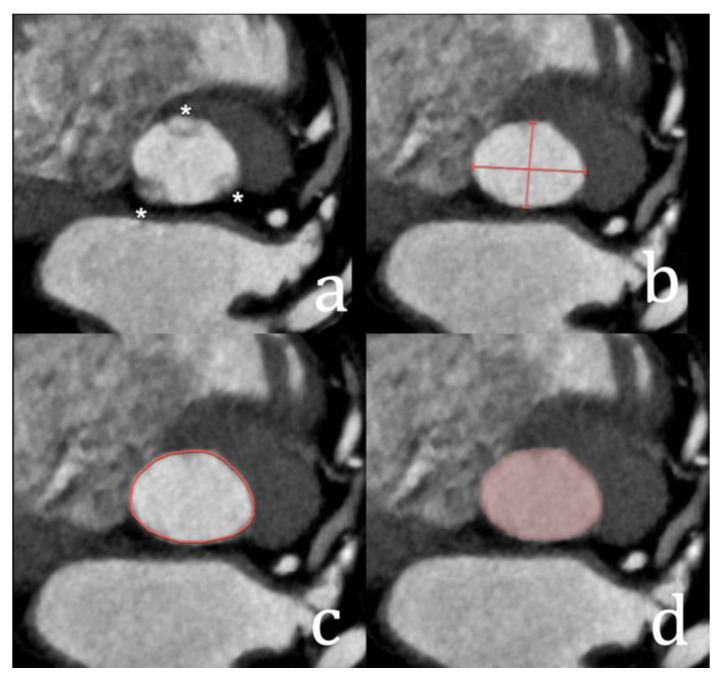
Recommended measurements at the level of the aortic annulus. Annular planimetry must be performed at the aortic annulus. Using double-oblique reformatted images, the correct plane lies just distally in the direction of the LVOT from the image where the leaflets can just barely be seen (asterisk in (**a**)). In most cases, the aortic annulus will have in cross-sectional images an oval shape, with short and long axes (red lines in (**b**)). Additional measurements are the annular perimeter (**c**) and annular area (**d**), marked in red.

**Figure 6:**
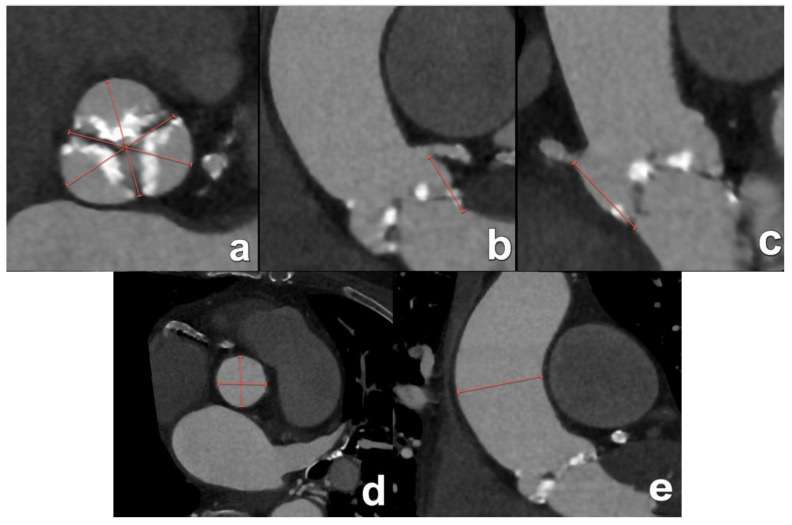
Besides the aortic annulus, a number of other measurements (marked with red lines) are considered of importance to determine patient eligibility and optimal matching of the patient’s anatomy to a specific device size. These include the cross-sectional dimensions of the aortic sinus (**a**), the distances from the annular plane to the ostium of the left main coronary artery (**b**) and the right coronary artery (**c**), the dimensions of the sinotubular junction (**d**) and the maximum double-oblique diameter of the ascending aorta (**e**).

In practice, measurements start with correctly aligning the imaging plane perpendicular to the long axis of the aortic annulus and aortic sinus. This implicates a correctly position double-oblique plane, for which the volumetric dataset of CT is especially suited. Detailed instructions on how to perform this plane reformation can be found elsewhere [14]. Consecutively, the most commonly performed measurements at the level of the aortic annulus are the short and long axes of the aortic annulus, the annular perimeter and the annular area (Figure 5). Other measurements that can be performed are presented in Table 3, and some are illustrated in Figure 6.

It is recommended to perform all measurements of the aortic annulus in the systolic phase, as this yields, in theory, the largest dimensions. However, as image quality may be suboptimal for various patient-related and technical reasons, it is recommended to obtain a correct measurement in a different phase (e.g., diastolic phase) rather than to force a suboptimal measurement in the systolic phase. This approach also implies that in TAVR candidates, an ECG-triggered protocol must be used that covers both phases of the cardiac cycle.

A proper match between the patient and a specific THV size will not only reduce potential complications during the procedure but also minimise post-procedural complications. One of the most prognostically important complications is the occurrence of paravalvular regurgitation (PVR), with blood leaking between a deployed THV and the wall of the aortic sinus. While the occurrence of this condition is multifactorial, an incorrect THV size (usually too small for the patient’s anatomy) has been recognised as an important factor. Even moderate PVR has important prognostic implications [16].

**Figure 7:**
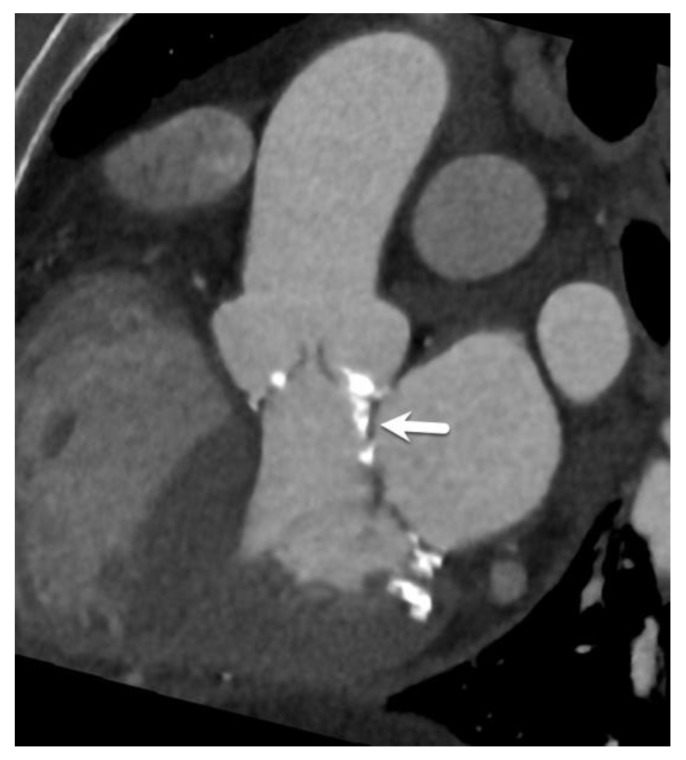
Prominent subvalvular calcification reaching over the anterior mitral valve leaflet (arrow). Subvalvular calcification can be encountered with varying degrees of severity and can lead to less stable device deployment. As such, its presence and amount must always be commented on.

It is important in this respect to perform at least a visual assessment of the severity of leaflet calcifications. During deployment of the THV, leaflet calcifications are crushed between the THV and the aortic sinus wall, where they may locally hamper device deployment. In the same context, the presence of subvalvular calcifications is an important point to mention in the radiology report (Figure 7).

**Figure 8:**
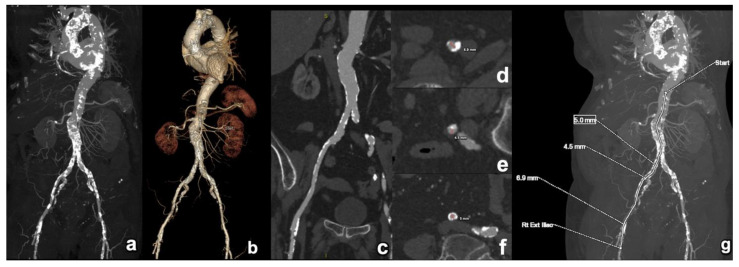
Assessing the patency route for the transfemoral delivery of an aortic THV. Initially, a comprehensive view of the thoraco-abdominal aorta is presented, utilising both maximum intensity projection (MIP) (**a**) and volume rendering (VR) techniques (**b**). This enables a global evaluation of the calcification load and distribution, most effectively visualised in the MIP image (**a**), while vascular tortuosity is better illustrated on the VR images (**b**). Furthermore, a curved multiplanar reformation (cMPR) of each iliac axis is acquired (only the right side is depicted here) for optimal visualisation of the lumen (**c**). Cross-sectional images (**d**–**f**) taken perpendicularly to this centreline provide an accurate evaluation of the true luminal diameter (red lines), which is essential for transporting the THV. In every segment of the arterial access route, identifying the narrowest luminal diameter is crucial, as localised narrowing can hinder device passage and increase procedural complexity and complication rates. In this instance, the right external iliac artery and the common iliac artery have focal minimal luminal diameters of 4–5 mm, rendering them less suitable for device transport compared to the contralateral side, which exhibits better patency. An overview of the narrowest luminal diameter sites is also provided (**g**). THV: transcatheter heart valve.

**Figure 9:**
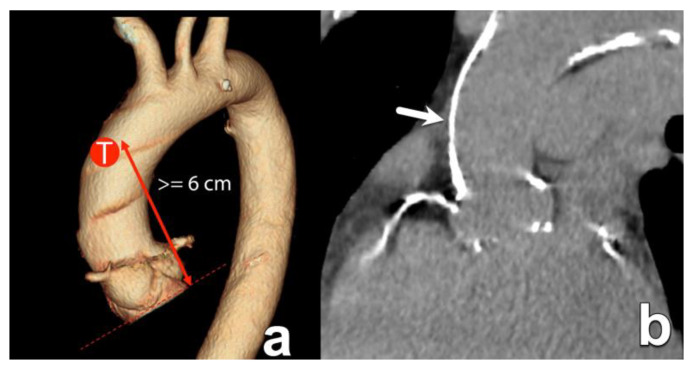
One of the possible access routes for both balloon- and self-expandable THVs is the transaortic approach. This approach, however, requires a clear landing zone in the anterior wall of the ascending aorta at about 6 cm above the annular plane (**a**). Panel (**b**) shows extensive wall calcification in an 86-year-old man with unavailable femoral access due to severely stenotic iliac arteries. In this patient, the transaortic approach is also precluded by the aforementioned very extensive calcifications in the aortic wall (arrow). The investigation of any possible obstruction along the possible access routes is paramount for procedural success, not only for the evaluation of route patency but also for determining patient eligibility to receive the device.

A second important task of CT is to evaluate the patency of the different access routes. While the endovascular approach using the groin femoral artery remains the access route of choice (Figure 8), other endovascular routes have been developed (Figure 9). Additionally, balloon-expandable valves also offer the possibility of a transapical transport. Over time, different access routes have been further developed. An overview is provided in Table 4.

Finally, while AS is in many instances an echocardiographic diagnosis, CT can be helpful in selected cases. Previous papers have highlighted the possibility of performing planimetry of the aortic valve area, showing a good correlation with transoesophageal measurement in patients with aortic stenosis [17,18]. Nevertheless, this method is, in practice, seldom used. In recent years, several studies have emerged indicating that the Agatston score, typically used to quantify coronary artery calcifications, can also be applied to quantify aortic leaflet calcifications derived from non-contrast computed tomography. It has since emerged as a valuable diagnostic tool in select cases of aortic stenosis (AS) where echocardiographic findings are discordant or inconclusive, often in cases with a low-flow, low-gradient configuration [19,20,21]. In such instances, quantifying aortic leaflet calcification using the Agatston method on non-contrast CT can provide supplementary diagnostic information beyond conventional clinical and echocardiographic assessments following ESC guidelines [6,22]. Table 5 provides an overview of current ESC guidelines indicating the likelihood of severe aortic stenosis depending on the degree of aortic leaflet calcification [22]. Note the sex difference in required valve calcifications for severe aortic valve stenosis [23]. According to some authors, this may be, in part, the result of women having more fibrotic components, resulting in a similar degree of aortic stenosis with a smaller calcium load [24]. A detailed explanation of how to quantify aortic leaflet calcification is beyond the scope of this paper, although several excellent review papers exist on this topic [20]. It is important to note that only leaflet calcifications should be included in the total Agatston score of calcification. Calcifications in the left ventricular outflow tract, aorta, mitral annulus and coronary arteries should not contribute to the total calcium load assessment [20].

## 4. Mitral Valve Repair and Replacement

Mitral valve disease encompasses a diverse set of valvular pathologies, which, similarly to aortic stenosis, have an increasing incidence in the older population and consequently affect millions of individuals worldwide. The vast majority of mitral valve disease consists of mitral valve regurgitation, which can be primary or secondary; these are to be considered two distinct diseases with different natural histories, pathophysiological mechanisms of MR and responses to surgical repair [25].

The primary indication for transcatheter treatment in mitral valve disease is severe symptomatic mitral regurgitation in patients, in analogy to TAVR, who are at high or prohibitive surgical risk due to a concomitant comorbidity. Other candidates include patients with chronic severe primary or secondary mitral regurgitation at high surgical risk who remain refractory to optimal medical therapy.

The mitral valve apparatus has some different key differences from the aortic valve, influencing the possible therapeutic options. Besides the difference in cuspidity (tricuspid vs. bicuspid), an important distinction is the D-shaped asymmetric mitral annulus, which contrasts with the more oval but symmetric aortic annulus. Additionally, the mitral valve has a complex subvalvular component composed of chordae tendineae and papillary muscles and extends into the LVOT [26,27].

Currently, both transcatheter mitral valve repair (TMVr) and replacement (TMVR) are available. However, in contrast to techniques for the aortic valve, TMVr is often preferred and more widely available; TVMR is currently only available in selected specialised centres [28,29]. Several factors are responsible for this discrepancy. TMVr has the benefit of preserving the native valvular anatomy and subvalvular apparatus, which helps in maintaining left ventricular geometry and function. Also, given the D-shaped mitral annulus extending into the LVOT, TMVR carries a higher risk of LVOT obstruction. This obstruction risk is lower in TMVr, especially in patients with an intact anterior mitral leaflet [30]. Finally, there is a significant accumulation of clinical evidence for TMVr using, e.g., the Mitraclip device (Abbott, Abbott Park, Illinois, USA), documented in clinical trials, demonstrating an adequate safety profile, application in a wide range of patients and a lower thrombosis risk. Nevertheless, developments in TMVR are rapidly evolving, and this technique can still be preferred in cases of severe structural valve damage, some forms of functional mitral regurgitation, severe mitral annular calcifications and failed previous repair [28,31].

An overview of common measurements required when using the Mitraclip device is provided in Table 6. It is important to remember that all these measurements have to be interpreted in conjunction with echocardiography data for optimal assessment.

**Figure 10:**
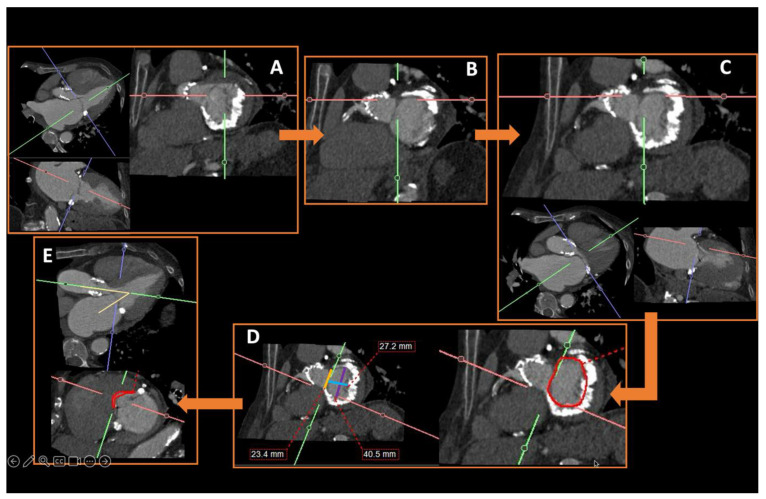
Useful pre-planning measurements to obtain before pre-transcatheter mitral valve repair in an 80-year-old female with a heavily calcified mitral annulus. The first step (**A**) is to create a short-axis view by cutting through the apex of the left ventricle and then identifying the left fibrous trigone (**B**) on the short axis. From there, the cross-hair is aligned along the right fibrous trigone (**C**) on the long axis to finally obtain the mitral annulus plane (**D**), from which the following measurements are taken: trigone–trigone diameter (yellow line), septo-lateral distance (blue line), inter-commissural distance (purple line) and area of the annulus (red circle). Finally, to identify the neo-LVOT, the shortest distance on the LVOT view between the septal leaflet of the mitral valve and the LVOT is determined (**E**), against the plane from which the aortomitral angle can be measured (yellow lines). Perpendicular to this level, the neo-LVOT area will be contoured in the short-axis view (red circle). Here, the angle was 44°, which indicates a low risk of neo-LVOT obstruction (risk increases when the angle approaches or exceeds 90°). The area was <190 mm^2^, indicating a high risk of neo-LVOT obstruction, therefore unfavourable for transcatheter mitral valve repair. Additional parameters that can be obtained in this view are the aortomitral length (increased risk if >30 mm) and the maximum myocardial septal thickness (increased risk if >14 mm in end-diastole).

**Figure 11:**
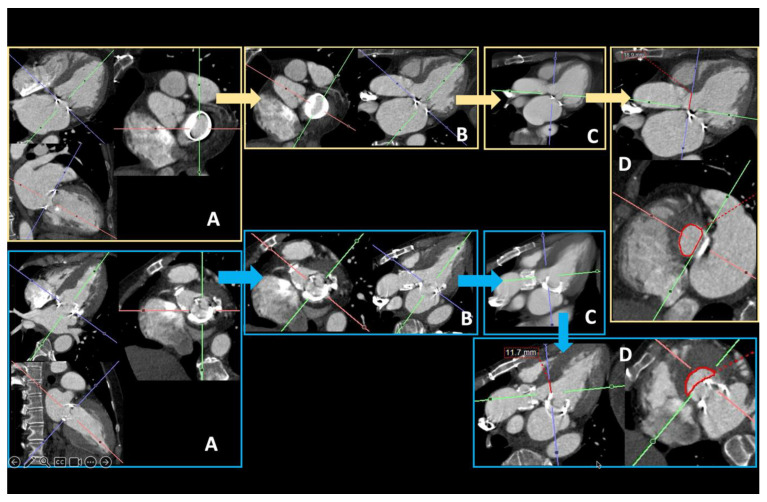
Useful pre-planning measurements to provide before pre-transcatheter mitral valve repair after prior mitral valve repair: valve in ring (yellow contours, upper line) and valve in valve (blue contours, bottom line). The steps to determine the neo-LVOT are similar: First, (A) obtain a short-axis view of the valve plane by cutting through the apex of the left ventricle on the long axis. From the centre of the valve plane, rotate the cross-hair through the LVOT on the short axis to obtain a long-axis LVOT view (B). To easily identify the most inferior point of the septal mitral valve leaflet, we suggest increasing slab thickness (C), and from there, position the centre of the cross-hair at this point and rotate the cross-hair to cut through the LVOT to obtain the neo-LVOT on the short axis (D). For the long-axis LVOT (back to thin slice), provide the annulus-to-septum distance (red line) and the neo-LVOT area (red circle). An annulus-to-septum distance of <18 mm indicates a higher risk of neo-LVOT obstruction.

An approach to a pre-procedural assessment using CT in a patient before TMVr is illustrated in Figure 10. For TMVr after previous mitral valve repair, Figure 11 illustrates common reformations and measurements.

While these measurements are used to determine patient eligibility and procedural feasibility, other CT-derived measurements have prognostic value. Mitral valve annular area (MAA) can be assessed with CT, as a smaller MAA and smaller anterio-posterior and medial–lateral annular diameters are associated with higher residual transmitral pressure gradients after the procedure and as such can potentially predict residual post-procedural mitral stenosis [32]. Left atrial dimensions are a particular feature in patients with both primary and secondary MR as a consequence of volume overload and increased filling pressures, respectively [25]. Recent evidence suggests that left atrial dilatation is an independent and strong predictor of adverse long-term outcomes after TMVR, but this does not seem to influence technical success or procedural failure [33].

## 5. Transcatheter Tricuspid Valve Replacement

Contrasting with non-surgical mitral and aortic valve treatments, transcatheter approaches for tricuspid valve (TV) disease are currently less prevalent, and evidence for their widespread application is still being gathered. A detailed discussion is therefore beyond the scope of this paper.

Currently, these approaches is reserved for selected patients requiring treatment for severe tricuspid regurgitation (TR) who are at high surgical risk [34]. This is, however, likely to become increasingly relevant, as the number of patients with TR is increasing in the Western world. TR is being recognised as having adverse long-term clinical consequences, and surgical interventions are associated with significant morbidity and mortality [34,35]. The majority of TR cases are secondary to pulmonary hypertension and left-heart disease.

The anatomy of the TV poses particular challenges for transcatheter interventions. As with the mitral valve, the tricuspid annulus has a non-planar, three-dimensional configuration. In patients with severe TR, it nevertheless becomes more dilated, circular and flattened. Furthermore, its size can significantly change depending on the chosen phase of the cardiac cycle, increasing the risk for paravalvular regurgitation [36]. The tricuspid annulus also does not calcify, making it more challenging to achieve post-procedural stability of the THV in a non-calcified landing zone [37]. Some additional important anatomical relationships exist, including the proximity of the TV to the right coronary artery (important for annuloplasty-based therapies), the coronary sinus, the AV node in the triangle of Koch and the sometimes-steep angulation of the TV with the superior and inferior venae cavae, the latter which can complicate transcatheter device delivery [34,37,38].

CT can help in the pre-procedural assessment by providing accurate planimetry of the tricuspid annulus, most commonly the area, perimeter and anteroposterior/septolateral diameters [37,39]. Like with all other cardiac valves, THV sizing algorithms remain device specific. Other important information provided by CT includes the dimensions and function of the right ventricle and a detailed review of the mentioned critical anatomical relationships [40,41]. Finally, similarly to TAVI, for example, CT can help by suggesting optimal fluoroscopic angles for device implantation and by evaluating the patency of the vascular access route.

## 6. Transcatheter Left Atrial Appendage Occlusion

Atrial fibrillation (AF) is a common condition in which fibrillation of the wall replaces the normal contractility of the left atrium. This abnormal wall motion, which lacks the normal atrial systole and diastole, not only causes the immediate loss of atrial function but also severely slows the normal circulation of blood in the left atrium [42]. The left atrial appendage (LAA), with its unique and variable anatomical configuration, is particularly susceptible to circulatory stasis and thrombus formation, responsible in 91% of cases for the thrombus source [43]. This is important, as AF-related thromboembolism exhibits a frequency of about 5% per year and is associated with a higher morbidity and mortality than stroke caused by other conditions [42].

While stroke risk is often treated with oral anticoagulants, some patients are not well suited for the long-term use of this therapy due to a prohibitive risk of bleeding or a concomitant condition preventing chronic oral anticoagulant administration. As for selected patients who are at an increased risk for thromboembolism, transcatheter exclusion of the LAA from the circulation is, for these patients, a viable therapeutic option.

While echocardiography is commonly used in the pre-procedural work-up of these patients, CT has significant benefits as a true 3D imaging modality in the detection of procedural contraindications, the evaluation of the native LAA anatomy and the planning of the procedure [44].

Some conditions are considered important contraindications for the procedure. Absolute indications include the presence of intracardiac thrombus, specifically in the left atrium or LAA, active cardiac inflammation, the inability to tolerate anticoagulation therapy after the procedure and known hypersensitivity to the device material.

As previously explained, the LAA is a predilection site for developing atrial thrombus in patients with AF, given its prominent trabeculation, thin wall and multilocular configuration. This is important, as the presence of a pre-existing LAA thrombus is an absolute contraindication for LAA closure.

**Figure 12:**
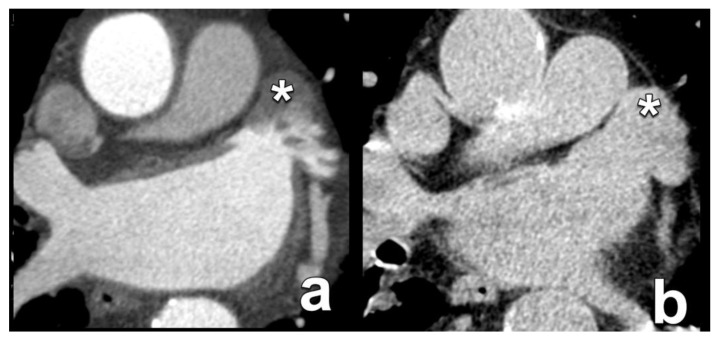
Benefit of an additional delayed-phase image acquisition to distinguish between slow flow and a true thrombus in the LAA. The initial acquisition in the arterial phase shows the incomplete opacification of the LAA (asterisk in (**a**)). However, an additional delayed-phase acquisition shows homogeneous opacification, excluding the presence of a thrombus (asterisk in (**b**)). This is important, as an LAA thrombus is a contraindication for LAA closure and represents an increased risk for thromboembolic stroke. LAA: left atrial appendage.

While thrombus is usually evaluated with echocardiography, CT can also be used to detect or exclude the presence of thrombus in the LAA [45,46]. However, given the slower opacification of the LAA compared with the rest of the atrium, the usual arterial-phase-only scan protocol must be modified to include a delayed phase to distinguish between an incompletely opacified LAA with a pseudo-thrombus appearance and a true clot formation (Figure 12). While different delay times have been suggested by different authors (ranging between 30 and 180 s), a recent expert consensus document recommends a 60 s delay from the contrast peak detected by the bolus tracking technique [43]. General features of an LAA thrombus are a Hounsfield density (HU) < 100 and the persistence of delayed-phase imaging. Other techniques for thrombus detection have been reported, including scanning with the patient in a prone position, the use of spectral imaging techniques and a split-bolus or double-contrast injection. However, these are seldom routinely used in practice due to their practical repercussions.

Pericarditis also increases the risk of procedural complications, as inflammation makes the atrial tissue more prone to injury, bleeding and possible perforation [43]. Furthermore, adhesion caused by chronic pericarditis can increase procedural complexity and risk, locally distorting LAA anatomy. The presence of pericardial effusion can also interfere with peri-procedural imaging, increasing the risk of complications. Finally, performing the procedure on inflammatory tissue will not only lead to slower healing but also carries a risk of exacerbating pericarditis.

**Figure 13:**
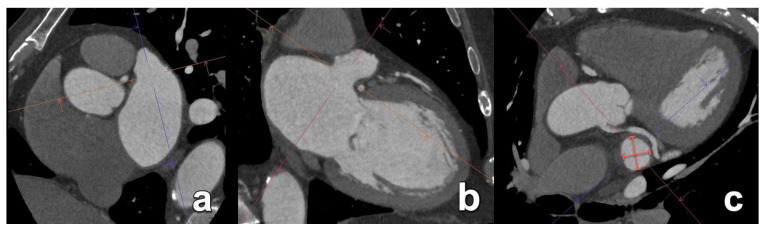
Multiplanar image reformatted to obtain the correct imaging plane for planimetry of the LAA. This manipulation is typically performed at a workstation, where the imaging cross-hairs need to be aligned with the left upper pulmonary vein and the left circumflex artery (**a**,**b**). The resulting image (**c**) then provides a double-oblique view of the base of the LAA, which can then be used for measurement of the ostium of the LAA (red lines). LAA: left atrial appendage.

One of the most common indications for CT before LAA closure is the evaluation of the LAA anatomy, specifically the planimetry of the LAA orifice, the device landing zone and the depth of the LAA. It is important to note that all measurements must follow the manufacturer’s guidelines to achieve maximum procedural success be and performed on properly reformatted images (Figure 13). While many devices exist, the Amplatzer Amulet (Abbott, USA) and Watchman (Boston Scientific, USA) devices are among the most popular (Table 7). The definition and position of the landing zones are device dependent, but the procedure to acquire these is similar. Other typical dimensions to acquire are the orifice and the depth of the appendage.

**Figure 14:**
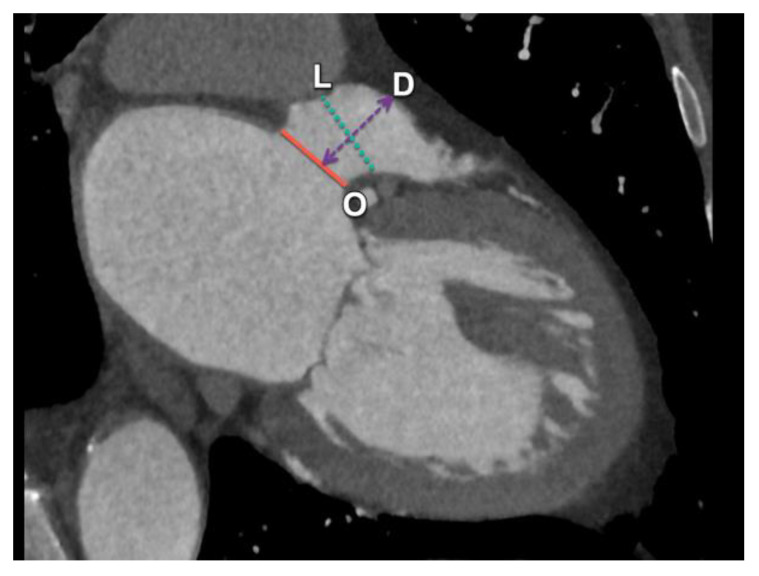
Illustration of LAA planning before the deployment of an Amulet LAA closure device. After correctly reformatting the CT acquisition (see Figure 11), three essential landmarks must be evaluated. The LAA orifice (O) is for this device formed by a line connecting the left upper pulmonary vein ridge and the left circumflex artery (red line). The depth of the LAA (D) is measured perpendicularly from this orifice to the roof of the LAA (dotted lines with arrows). The landing zone (L, green dotted line) for an Amulet device is located about 10–12 mm from the orifice, following a trajectory perpendicular to the LAA wall. LAA: left atrial appendage.

Sizing algorithms are manufacturer dependent, with the maximum diameter of the landing zone often used as the criterion to select a specific device size (Figure 14). This must be interpreted cautiously, as guidelines are based on echocardiography-derived measurements. We routinely also provide CT-based area and perimeter measurements of the landing zone, although more studies are required to investigate the additional value of these measurements.

Finally, the atrial septum must be evaluated for any obstructive pathology that may hamper the passage of the catheter from the right to the left atrium.

## 7. CT Before Pulmonary Vein Ablation

Pulmonary vein (PV) ablation, also known as pulmonary vein isolation, is a technique to treat AF, the most common sustained cardiac arrhythmia. AF arises from the pulmonary veins: not only do the PVs’ myocardial sleeves present cells with electrical automaticity, resulting in ectopic beats that initiate AF, but they also offer an adequate architectural and electrophysiological environment to maintain AF [47]. Therefore, the strategy to control AF is to target the PV.

To achieve this goal, both surgical and percutaneous catheter-based ablation techniques have been proposed. The most validated surgical ablation method is the MAZE procedure. However, this requires invasive surgery. On the other hand, the catheter-based ablation technique can achieve similar results through a minimally invasive approach. Therefore, catheter-based ablation is the preferred option when no other concomitant cardiac surgery is required. It is also considered the first-line option in patients with paroxysmal or persistent AF that is resistant or intolerant to antiarrhythmic drug therapy [48]. Minimally invasive surgical approaches have been developed, but they should be considered only after a failed catheter-based ablation.

**Figure 15:**
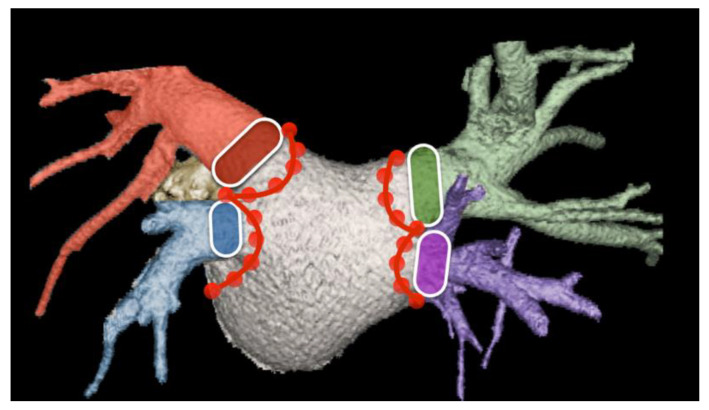
Volume-rendered CT image of the left atrium and pulmonary veins. The four pulmonary veins can be seen draining into the left atrium. The lined oval structures indicate that the myocardial sleeves contain potential arrhythmogenic cells. The principle of this treatment technique is the electrical isolation of these myocardial sleeves from the rest of the left atrium by catheter-induced scar tissue surrounding the ostia of the pulmonary veins (dotted right lines). Therefore, it is important to be aware of any anatomical variation in order to isolate all draining veins.

Percutaneous catheter-based ablation traditionally involves delivering a radiofrequency current from the catheter tip to create a thermal injury (although cryoablation has been recently developed). The catheter is inserted through the venous system, usually via femoral access, and is advanced to the right atrium before gaining access to the left atrium (LA) through a trans-interatrial septum puncture or a patent foramen ovale. Once in the LA, a 3D electrical map is generated through the catheter to identify sources of arrhythmia, with subsequent ablation of those sources performed by delivering an electrical current, usually around the four PV ostia (Figure 15). If necessary, this ablation can be extended to the left atrium wall based on information delivered by the 3D map.

It is therefore crucial to have a clear understanding of the precise anatomy of the pulmonary veins, as well as the right and left atria, any potential anatomical variations and nearby mediastinal structures. This understanding is essential for pre-procedural planning in order to improve accuracy, reduce procedure time and patient exposure to radiation and minimise complications. CT is the preferred imaging method for this pre-procedural evaluation.

There are usually four PVs, two on each side of the LA: two superior veins and two inferior veins draining into the LA. The right superior vein drains the right upper lobe and right middle lobe, the right inferior vein drains the right lower lobe, the left superior vein drains the left upper lobe and lingula and the left inferior vein drains the left lower lobe. It is worth noting that the left superior PV alone accounts for half of all ectopic beats leading to AF.

**Figure 16:**
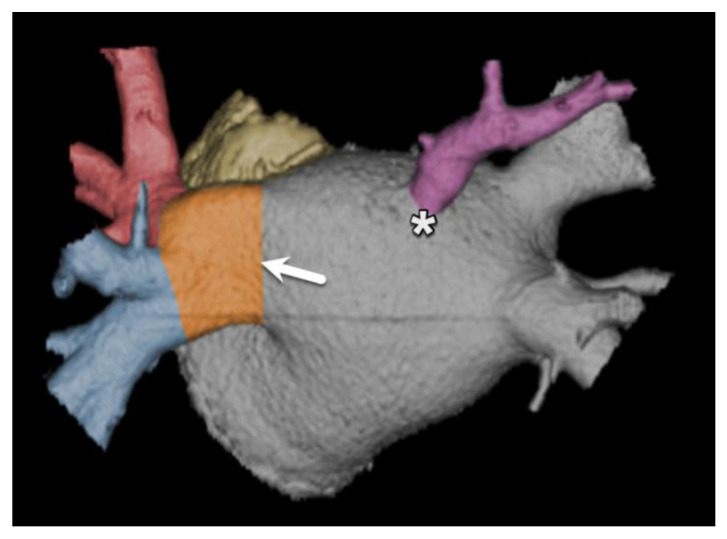
Volume-rendered CT image of the left atrium and pulmonary veins with anatomical variations. Both a common drainage (common trunk) of the left pulmonary veins (arrow) and a contralateral accessory pulmonary vein (asterisk) can be seen.

Anatomy variations like a common trunk or a supernumerary PV can be present (respectively seen in up to 25% and 19% of individuals); the CT report should therefore mention the number of PV ostia (defined as the atrio-pulmonary venous junction) into the LA to make sure that all PVs are treated (Figure 16) [49]. Also, any anomalous pulmonary venous return (most frequently draining into the superior vena cava) should be mentioned.

The report should also mention the size of the PV ostia as well as the length of the PV before the first-order PV branch, as smaller ostia and shorter lengths indicate a higher risk of post-ablation PV stenosis.

Multiplanar reconstructions are helpful to better assess the anatomy. Possible post-processing with 3D volume rendering in multiple projection angles is encouraged to offer “angiographic views” [50]. Finally, comments on severe angulation of the PV are welcome to guide the operator.

Atrial remodelling with atrial enlargement is known to promote chronic AF. An LA anteroposterior diameter of ≥50 mm for men and ≥45 mm for women can be used as a visual threshold to suggest LA enlargement [51]. A thrombus (even more in an enlarged LA) in the LA and left atrial appendage (LAA) should be looked for as an absolute contraindication to catheter ablation. A further description of the LAA (including size, ostium dimensions, shape) should be provided for patients undergoing cardiac surgery with AF to guide concomitant surgical LAA closure [48] but are beyond the scope of this paper.

The documentation of any other relevant cardiac or chest abnormality (for example lung neoplasm on lung windows) is warranted. Comments on the presence of pericardial effusion should be made. It is important to obtain a baseline, as the identification via transoesophageal echocardiography of new pre-procedure pericardial effusion raises the suspicion of hemopericardium and possible cardiac perforation. Finally, the oesophageal course should be mentioned in the report, indicating the risk of oesophageal injury, from simple ulcers (up to 47%) to the most severe but rare LA–oesophageal fistula, which can be increased in the case of close contact between the oesophagus and the PV [52].

Although catheter ablation for pulmonary vein isolation has a success rate of over 90%, it is associated with several potential complications. These complications include thromboembolic events such as strokes, hemodynamically significant pulmonary vein stenosis after the ablation procedure, oesophageal perforation, haemopericardium, haemothorax and significant arrhythmias, including asystole, which can occur during the procedure due to the stimulation of the vagus nerve fibres located within the pulmonary vein walls. The recurrence of atrial fibrillation can also occur due to inflammation at the border of the central necrosis. In such cases, repeat catheter ablation remains a viable option.

Important parameters to check and comment on are summarised in Table 8.

## 8. Conclusions

Advanced imaging techniques, particularly CT, have become integral to the planning and execution of various transcatheter cardiac interventions. These modalities provide crucial pre-procedural information for different interventions, as explained in this paper, and their number is growing. The detailed anatomical data obtained through these imaging methods allow for the precise measurement of target structures, evaluation of access routes and identification of potential complications. This information is essential for appropriate device selection, procedural planning and risk assessment.

As transcatheter techniques continue to advance, the role of sophisticated pre-interventional imaging is likely to become even more critical in ensuring procedural success and optimising patient outcomes. As such, an important role is reserved for the attending radiologist, playing a central role not only in the imaging of the involved patients but also as a key figure in clinical patient care.

## Figures and Tables

**Table 1:** Overview of congenital abnormal interatrial communications. Note that sinus venosus and coronary sinus defects are not considered true ASDs, although both lead to interatrial communications. A PFO is a normal foetal communication that may persist into adulthood, allowing for potential right-to-left shunting under certain conditions.

Type of Interatrial Communication	Prevalence	Location	Associated Anomalies/Syndromes	Standard of Treatment
Ostium secundum ASD	70–80%	Fossa ovalis of the atrial septum	More common in females (65%–70%)	Endovascular closure in suitable anatomy
Ostium primum ASD	10–15%	Near the atrioventricular valves	Cleft mitral valve, partial atrioventricular septal defects, Down syndrome, DiGeorge syndrome, Ellis Van Creveld syndrome	Surgery
Sinus venosus ASD	5–10%	Deficiency of the common wall between the SVC and the right-sided pulmonary veins	Anomalous pulmonary venous connection	Surgery
Coronary sinus septal defect (unroofed coronary sinus)	1%	Incomplete formation of the left arteriovenous fold	May be associated with a left-sided SVC	Surgery
Patent foramen ovale (PFO)	25–30% of the general population	Fossa ovalis of the atrial septum	Cryptogenic stroke, migraine with aura, decompression sickness in divers	Usually no treatment required

**Table 2:** Overview of different septal closure devices. Almost all closure devices require an atrial septal rim of at least 5 mm for device stabilisation.

Device	Maximum Defect Size	Minimum Rim Requirements	Other Considerations
Amplatzer Septal Occluder	Up to 38 mm	Generally 5 mm for all rims but can be used with deficient aortic rims	Avoid oversizing in deficient aortic rim cases
Occlutech Figulla Flex II	Up to 39 mm	Similar to the Amplatzer, generally 5 mm for all rims	May be more flexible in cases with deficient rims
Gore Cardioform Septal Occluder	Up to 18 mm	Requires adequate rims all around, especially in larger defects	Not suitable for large defects (>18 mm) evice size should be at least twice the defect size
CeraFlex ASD Occluder	Up to 40 mm	Similar to the Amplatzer, generally 5 mm for all rims	Flexible delivery system may help in difficult anatomies
Cocoon Septal Occluder	Up to 38 mm	Similar to the Amplatzer, generally 5 mm for all rims	Softer device may conform better to varying anatomies
Nit-Occlud ASD-R	Up to 28 mm	Requires adequate rims, especially for larger defects	“Reverse” configuration may help in some anatomies Not suitable for very large defects

**Table 3:** Overview of common measurements with CT in a TAVI candidate. TAVI: transcatheter aortic valve implantation. THV: transcatheter heart valve.

Measurement	Description	Comments
Aortic annulus dimensions	Long- and short-axis diameters Area Perimeter	Sizing algorithms are device- and manufacturer-specific.
Aortic valve cuspidity	Comment on number of leaflets	The presence of a bicuspid valve may increase procedural complexity and increase the risk for permanent pacemaker implantation after the procedure.
Coronary ostia heights	Distance from annular plane to bottom of left and right coronary ostia	As a rule of thumb, a minimum diameter of 10 mm is considered the threshold to avid ostial coronary obstruction by migrated leaflet calcifications.
Sinus of Valsalva dimensions	Long- and short-axis cross-sectional diameters Largest cross-sectional diameter Commissure-to-cusp diameter Height of the coronary sinus	The aortic sinus needs to be wide enough to accommodate displaced native leaflet calcifications during the deployment of the THV.
Sinotubular junction diameter	Diameter at the sinotubular junction	
Tube angulation	Angles of the tube where all basal insertion points of the aortic leaflets are aligned in one plane	Knowledge of this tube angulation can reduce procedure time and the use of contrast material.
Ascending aorta diameter and wall calcifications	Diameter at 40 mm above annulus Presence and severity of aortic wall calcification	For transaortic access, the landing zone is about 60 mm above the annular plane.
Left ventricular outflow tract and subvalvular calcifications	Long- and short-axis diameters	The presence of prominent subvalvular calcification may interfere with THV deployment.
Aortic valve calcification	Quantification and distribution of leaflet calcification	The Agatston score is used to quantify leaflet calcifications. This is not routine and typically reserved for low-flow low-gradient cases with inconclusive echocardiography results.
Left ventricular basal septum thickness	Presence of basal left ventricular hypertrophy	A hypertrophic basal septum may contribute to device instability.
Peripheral access vessel diameters	Minimal luminal diameters of the iliofemoral arteries	A minimal luminal diameter of 5–6 mm is generally required, depending on the delivery system.
Aortic angulation	Angle between the LVOT and ascending aorta planes	A steep angulation increases procedural complexity.

**Table 4:** Overview of potential access routes for device delivery in a TAVI candidate. TAVI: transcatheter aortic valve implantation.

**Access Route**	Description	Advantages	Disadvantages/Considerations
Transfemoral	Most common and preferred approach. Access is typically through the femoral artery.	Least invasive. Preferred when feasible.	Requires an adequate iliofemoral vessel size and a lack of severe tortuosity/calcification.
Transapical	Through the left ventricular apex via small chest incision.	Option when transfemoral route not possible. Direct access to the aortic valve.	Only applicable to balloon-expandable valves. More invasive. Requires a normal LV apex. A steeper angle may complicate procedure.
Transaortic	Direct access to the ascending aorta via mini-sternotomy.	Option when transfemoral route not possible.	More invasive. The amount/location of ascending aorta wall calcification is important, typically at about 6 cm above the annular plane.
Subclavian/axillary	Through the subclavian or axillary artery.	Alternative when transfemoral route not possible.	Left side preferred due to angulation.
Transcarotid	Through the common carotid artery.	Alternative when other routes not possible.	Less commonly used.
Transcaval	Through the inferior vena cava and across the abdominal aorta.	Option in severe peripheral vascular disease.	Complex technique. Calcification-free window in aorta needed. Seldom used.

**Table 5:** CT-derived Agatston score of aortic leaflet calcification and the likelihood of severe aortic stenosis (data from reference [22]).

Likelihood of Severe Aortic Stenosis *	Men	Women
Highly likely	>3000	>1600
Likely	>2000	>1200
Unlikely	<1600	<800

* Thresholds for severe aortic stenosis assessed by means of CT measurements of aortic valve calcification (Agatston score).

**Table 6:** Overview of CT measurements before the use of the Mitraclip device (Abbott, Abbott Park, Illinois, USA).

Measurement	Description	Relevance
Mitral valve anatomy	Leaflet morphology, scallop identification (A1–A3, P1–P3)	Determines suitability for edge-to-edge repair
Coaptation depth	Distance from the annular plane to the point of leaflet coaptation	Should be ≤11 mm for optimal results
Coaptation length	Extent of the leaflet overlap	Ideally ≥2 mm for successful clipping
Flail gap	Distance between the flail segment and the opposing leaflet	Should be ≤10 mm
Flail width	Width of the flail segment	Should be ≤15 mm
Mitral valve area	Planimeter area of the mitral valve orifice	Should be ≥4 cm^2^ to avoid post-procedure mitral stenosis
Tenting height	Height of leaflet tenting in functional MR	Affects the likelihood of successful repair
Leaflet calcification	Extent and location of calcification	Heavy calcification may preclude successful clipping
Intercommissural distance	Distance between the anterior and posterior commissures	Aids in determining the number of clips needed
Left atrial size	Dimensions and volume of the left atrium	Affects the procedural approach
Interatrial septum	Thickness and location of the fossa ovalis	Guides the trans-septal puncture site
Neo-LVOT assessment	Simulated LVOT after clip placement	Assesses the risk of LVOT obstruction
Subvalvular apparatus	Papillary muscle and chorda anatomy	Ensures no interference with clip deployment

**Table 7:** Overview of the Amplatzer Amulet and Watchman LAA closure devices size ranges. LAA: left atrial appendage.

Device	Available Device Sizes (mm)	Applicable LAA Ostium Sizes (mm)	Landing Zone Diameter
Amulet	16–34	11–31	12–15 mm distal to orifice
Watchman	21–33	17–31	10–20 mm distal to ostium
Watchman FLX	20–35	17–31.5	10–20 mm distal to ostium

**Table 8:** Overview of common topics to include in the radiology report before a PVI procedure. PVI: pulmonary vein isolation.

Pulmonary veins	Number: normal anatomy Orifice size Length of the PV to first division Anomalous pulmonary venous return Angulation
Left atrium	Size/volume Thrombus
Interatrial septum	Patent foramen ovale Other abnormality
Other cardiac chambers	Thrombus Any abnormality
Oesophagus	Course
Pericardium	Pericardial effusion
Chest	Relevant abnormality
